# Targeting Microglial Activation States as a Therapeutic Avenue in Parkinson’s Disease

**DOI:** 10.3389/fnagi.2017.00176

**Published:** 2017-06-08

**Authors:** Sudhakar R. Subramaniam, Howard J. Federoff

**Affiliations:** Department of Neurology, University of California, Irvine, Irvine, CAUnited States

**Keywords:** Parkinson’s disease, microglia, therapeutics, neuroinflammation, polarization

## Abstract

Parkinson’s disease (PD) is a chronic and progressive disorder characterized neuropathologically by loss of dopamine neurons in the substantia nigra, intracellular proteinaceous inclusions, reduction of dopaminergic terminals in the striatum, and increased neuroinflammatory cells. The consequent reduction of dopamine in the basal ganglia results in the classical parkinsonian motor phenotype. A growing body of evidence suggest that neuroinflammation mediated by microglia, the resident macrophage-like immune cells in the brain, play a contributory role in PD pathogenesis. Microglia participate in both physiological and pathological conditions. In the former, microglia restore the integrity of the central nervous system and, in the latter, they promote disease progression. Microglia acquire different activation states to modulate these cellular functions. Upon activation to the M1 phenotype, microglia elaborate pro-inflammatory cytokines and neurotoxic molecules promoting inflammation and cytotoxic responses. In contrast, when adopting the M2 phenotype microglia secrete anti-inflammatory gene products and trophic factors that promote repair, regeneration, and restore homeostasis. Relatively little is known about the different microglial activation states in PD and a better understanding is essential for developing putative neuroprotective agents. Targeting microglial activation states by suppressing their deleterious pro-inflammatory neurotoxicity and/or simultaneously enhancing their beneficial anti-inflammatory protective functions appear as a valid therapeutic approach for PD treatment. In this review, we summarize microglial functions and, their dual neurotoxic and neuroprotective role in PD. We also review molecules that modulate microglial activation states as a therapeutic option for PD treatment.

## Introduction

**FIGURE 1:**
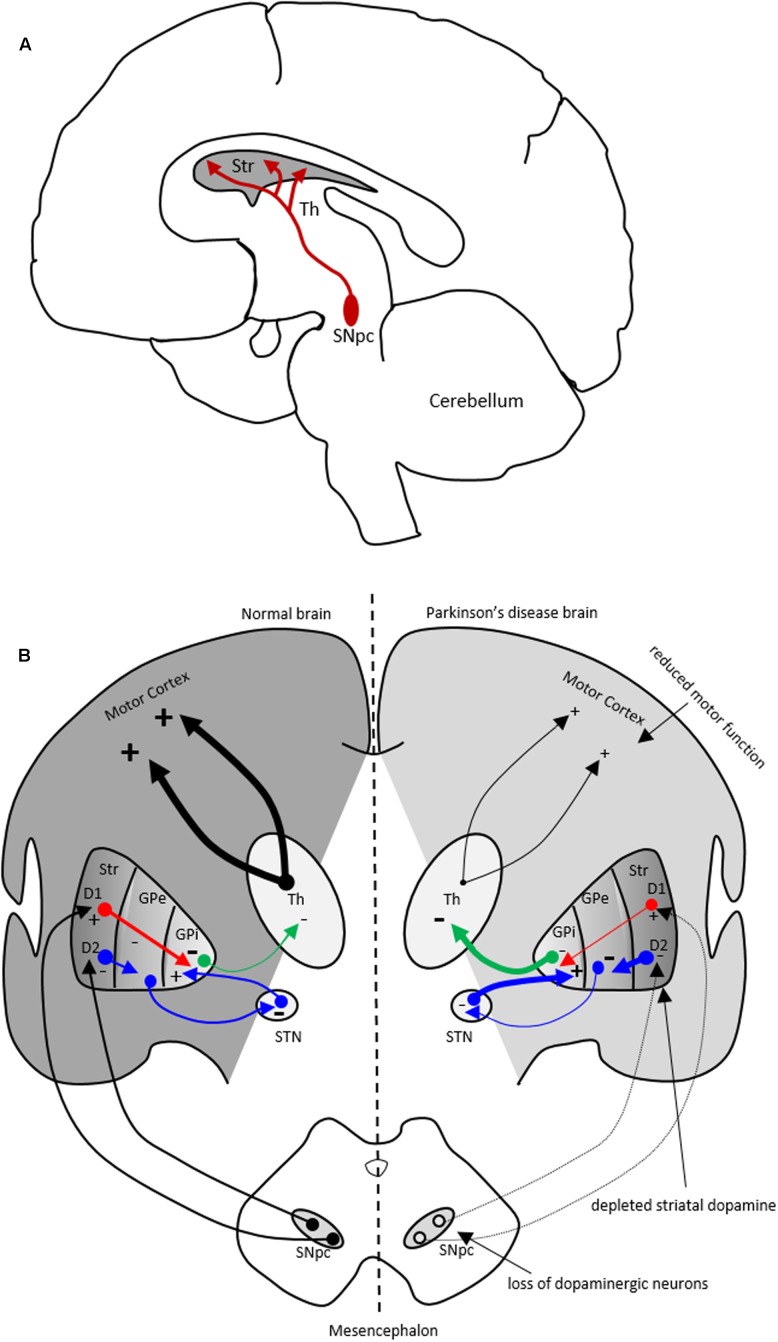
The nigrostriatal dopaminergic pathway and motor basal ganglia circuitry in Parkinson’s disease (PD) brain. **(A)** In the nigrostriatal pathway, the striatum, Str (caudate nucleus and putamen), receives dopaminergic innervation from the substantia nigra pars compacta (SNpc) in the midbrain. **(B)** A schematic showing normal motor basal ganglia circuitry (left side) and its irregularities in PD brain (right side), adapted from (Bjarkam and Sorensen, 2004). *Normal brain (left)*: The dopaminergic afferent neurons from the SN synapse with GABAergic neurons which display either D1 or D2 dopaminergic receptors. These GABAergic populations project directly (red arrows) or indirectly (blue arrows; through globus pallidus externa, GPe, and subthalamic nucleus, STN) to globus pallidus interna (GPi). The output from the GPi (green arrows) to the thalamus is inhibitory and modulates normal motor function. *PD brain (right)*: The loss of dopaminergic neurons in the SN and depletion of striatal dopamine leads to elevated inhibitory output from the GPi to the thalamus causing reduction in normal motor function. Inhibitory and excitatory inputs are marked as (–) and (+), respectively. The intensity of the inputs is marked with thickness of lines. Str, striatum; Th, thalamus.

Parkinson’s disease (PD) is a common movement disorder and the second most prevalent neurodegenerative disorder worldwide, that affects nearly 2% of the elderly population. PD is characterized by loss of dopaminergic neurons in the substantia nigra pars compacta (SNpc) and consequently reduced dopamine (DA) levels in the basal ganglia, causing motor dysfunction (**Figure 1**). Lewy bodies, intracellular proteinaceous inclusions, are the pathological hallmark within PD brains. Lewy bodies contain fibrillar α-synuclein among other proteins (Spillantini et al., 1997). It is evident that the immune system is involved in PD risk by genome-wide association studies (GWAS), implicating the human leukocyte antigen (HLA) locus with sporadic PD (Hamza et al., 2010) and, as well, by the neuropathology in PD brains demonstrating highly activated microglial and T-cells (McGeer et al., 1988; Imamura et al., 2003). Several inflammatory markers have been identified in SNpc of PD brains including cytokines and neurotrophins (Nagatsu et al., 2000; Hunot and Hirsch, 2003). Also, widespread microglial activation is a concomitant in PD neuropathology (Gerhard et al., 2006). A meta-analysis of anti-inflammatory drug trials revealed an association between non-steroidal anti-inflammatory drugs (NSAIDs) use and reduced risk for developing PD possibly implicating neuroinflammatory processes in the disease (Gagne and Power, 2010). Evidence supports the conclusion that microglia, the brain resident macrophage-like immune cells, participate in the inflammatory response of the disease (Qian and Flood, 2008; Long-Smith et al., 2009; Moehle and West, 2015). In addition, other observations implicate peripheral immune cells in PD (Saunders et al., 2012; Funk et al., 2013; Chen et al., 2015). Together these data indicate that inflammation and microglial activation contribute to the in pathogenesis of PD. Hence, immunomodulation might be a possible therapeutic avenue for PD.

The distribution of microglial M1/M2 phenotypes depends on the stage and severity of the disease. Understanding stage-specific switching of microglial phenotypes and the capacity to manipulate these transitions within appropriate time windows might be beneficial for PD therapy. In this review, we will outline different microglial activation states and provide evidence of M1/M2 activation states in PD. We will also discuss how manipulation of M1/M2 activation may be of potential therapeutic value.

## Microglia Functions

In the central nervous system (CNS), the innate immune response is predominantly mediated by microglia and astrocytes. Microglia play a vital role in both physiological and pathological conditions. Tissue-specific macrophages can be found in most tissues of the body, whereas microglia are present distinctly in the brain. Microglia are derived from primitive yolk sac myeloid progenitors that seed the developing brain parenchyma (Alliot et al., 1999; Ginhoux and Jung, 2014). Microglia represent 10–15% of the total population of cells within the brain and manifest different morphologies across anatomic regions (Lawson et al., 1990; Mittelbronn et al., 2001). Microglia appear to be involved in several regulatory processes in the brain that are crucial for tissue development, maintenance of the neural environment and, response to injury and promoting repair. Similar to peripheral macrophages, microglia directly respond to pathogens and maintain cellular homeostasis by purging said pathogens, as well as dead cells and pathological gene products (Gehrmann et al., 1995; Bruce-Keller, 1999; Stevens et al., 2007; Tremblay et al., 2010; Olah et al., 2011; Paolicelli et al., 2011). In addition, microglial function can be altered by interactions with neurons, astrocytes, migrating T-cells, and the blood–brain barrier itself (Gonzalez et al., 2014).

**FIGURE 2:**
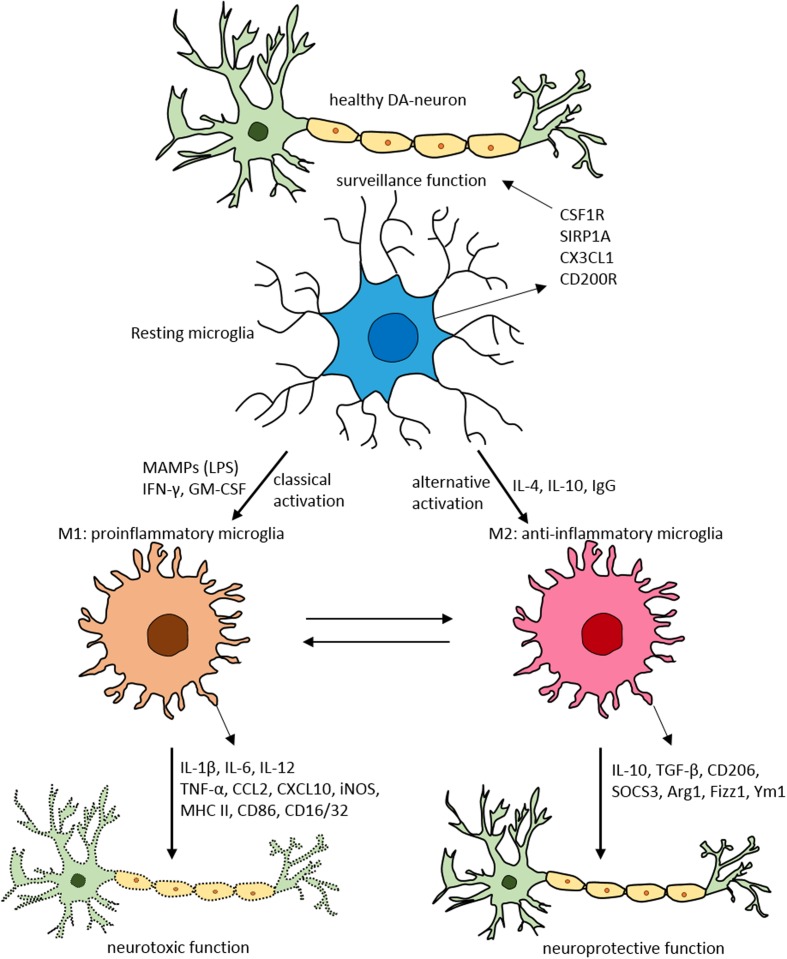
Schematic of microglial polarization states and function. In normal physiological conditions microglia acquire the surveillance phenotype to maintain all CNS cell types including neurons. To maintain this surveillance state, microglia secrete several factors including colony stimulating factor 1 receptor (CSF1R), signal regulatory protein CD172 (SIRP1A), chemokine CX3CL1 and CD200R. Upon classical activation when triggered by LPS, IFN-γ, or GM-CSF microglia acquire M1 pro-inflammatory phenotype leading to neurotoxicity by secreting several pro-inflammatory substances (for detailed list, see **Table 1**). When activated alternatively by IL-1, IgG, or IL-10 microglia attain M2 anti-inflammatory state prompting neuroprotection through secretion of variety of substances (for detailed list, see **Table 1**). Arg1, arginase 1; CCL, chemokine (C-C motif) ligand; CD, cluster of differentiation; CSF1R, colony stimulating factor 1 receptor; CXCL, chemokine (C-X-C motif) ligand; Fizz1, found in inflammatory zone; IL, interleukin; GM-CSF, granulocyte-macrophage colony-stimulating factor; IFN-γ, interferon-γ; iNOS, inducible nitric oxide synthase; LPS, lipopolysaccharide; MAMPs, microbe-associated molecular patterns; MHC-II, major histocompatibility complex II; SIRP1A, signal regulatory protein CD172; SOCS3, suppressor of cytokine signaling-3; TNF-α, tumor necrosis factor-α; Ym1, chitinase-like protein.

Under physiological conditions, microglia acquire a neural-specific, relatively inactive phenotype (Schmid et al., 2009) where they sample and inspect the local environment and other brain cells types (Davalos et al., 2005; Nimmerjahn et al., 2005). In a healthy brain, resting quiescent microglia exhibit a ramified morphology, with relatively long cytoplasmic protrusions, a stable cell body and little or no movement (**Figure 2**). Quiescent microglia extend processes into their surrounding environment (Nimmerjahn et al., 2005). This resting stage is partly maintained by signals conveyed by neuronal and astrocyte-derived factors (Neumann et al., 2009; Ransohoff and Cardona, 2010). The maintenance of this inactive state is regulated by several intrinsic factors like Runx1 (Runt-related transcription factor 1) and Irf8 (Interferon regulatory factor 8), and extrinsic factors such as TREM2 (triggering receptor also expressed on myeloid cells-2), chemokine CX3CR1 and CD200R (Kierdorf and Prinz, 2013). In the normal CNS environment, healthy neurons provide signals to microglia via secreted and membrane bound factors, such as CX3CL1, neurotransmitters, neurotrophins and CD22 (Sunnemark et al., 2005; Frank et al., 2006; Lyons et al., 2007; Pocock and Kettenmann, 2007). In addition, microglia express elevated levels of microRNA-124 which in turn reduces CD46, MHC-II (major histocompatibility complex II) and CD11b, to maintain the quiescent state (Conrad and Dittel, 2011).

## Microglial Activation: The Dual Roles of Microglia

As peripheral macrophages respond to endogenous stimuli promoting both pathogenic and protective functions, so do microglia. Upon exposure to endogenous stimuli microglia become activated. Among the gene products released by microglia are various substances including pro-inflammatory cytokines, neurotoxic proteins, chemokines, anti-inflammatory cytokines, and neurotrophic factors (Mahad and Ransohoff, 2003; Block et al., 2007; Benarroch, 2013; Nakagawa and Chiba, 2015). Microglia also display signaling immunoreceptors such as Toll-like receptors (TLRs), scavenger receptors (SRs), nucleotide binding oligomerization domains (NODs) and NOD-like receptors (Ransohoff and Brown, 2012). Fundamentally, the two polar states of microglia, the M1 and M2 phenotypes are associated phenomenologically with injury and homeostasis, respectively (as described below). Differential states of microglial activation within an injured tissue evolve during an inflammatory epoch (Graeber, 2010).

### M1 Polarization State

When classically activated, microglia acquire the M1 phenotype, characterized by pro-inflammatory and pro-killing functions that serve as the first line of defense. In M1 microglial activation state both secreted factors and cellular markers are dysregulated (**Table 1**). During M1 polarization (driving from another, often resting state), microglia release pro-inflammatory cytokines: interleukin-1β (IL-1β), IL-6, IL-12, IL-17, IL-18, IL-23, tumor necrosis factor-α (TNF-α), interferon-γ (IFN-γ) and nitric oxide, and chemokines: CCL2 (Mahad and Ransohoff, 2003; Kawanokuchi et al., 2006, 2008; Loane and Byrnes, 2010; Benarroch, 2013; Nakagawa and Chiba, 2015). Also upon M1 activation microglia appear to present phenotypic markers: inducible nitric oxide synthase (iNOS), cyclooxygenase-2 (COX-2), MHC-II, and CD86 (cluster of differentiation marker 86) (Chhor et al., 2013; Franco and Fernandez-Suarez, 2015) and, other substances including reactive oxygen species (ROS), reactive nitrogen species, and prostaglandin E2 (Benarroch, 2013; Nakagawa and Chiba, 2015). These orchestrated processes are to purge foreign pathogens and polarize T-cells to elicit an adaptive immune response.

**Table 1:** Microglial polarization states and substances produced.

Activation type/function	Source	Substances produced	Reference
M1 (classical activation): pro-inflammatory and pro-killing	LPS, IFN-γ	*Cytokines*: IL-1β, IL-6, IL-12, IL-17, IL-18, IL-23, TNF-α	Mahad and Ransohoff, 2003; Kawanokuchi et al., 2006 Kawanokuchi et al., 2008; Loane and Byrnes, 2010; Benarroch, 2013; Chhor et al., 2013; Franco and Fernandez-Suarez, 2015; Nakagawa and Chiba, 2015
		*Markers*: CD86, MHC-II	
		*Chemokines*: CCL2	
		*Metabolic enzyme/redox molecules*: iNOS, COX-2, reactive oxygen species and reactive nitrogen species prostaglandin E2	

M2a (alternative activation): tissue repair and phagocytosis	IL-4, IL-13	*Cytokines*: IL-10	Mahad and Ransohoff, 2003; Loane and Byrnes, 2010, Benarroch, 2013; Chhor et al., 2013; Franco and Fernandez-Suarez, 2015; Nakagawa and Chiba, 2015
		*Markers*: CD206, SR-A1, SR-B1, Arg1, Ym1, Fizz1	
		*Others*: extracellular matrix proteins, PPAR	
M2b (alternative activation): recruitment of regulatory T cells	Fcγ receptors, TLRs and immune complexes (IgG)	*Cytokines*: IL-1β, IL-6, IL-10, TNF-α	
		*Markers*: CD86, MHC-II	
		*Others*: SOCS3, COX-2, Sphk	
M2c (alternative activation): anti-inflammatory and healing	IL-10, TGF-β and glucocorticoids	*Cytokines*: IL-10, TGF-β	
		*Markers*: CD163	


*Arg1, arginase 1; CCL, chemokine (C-C motif) ligand; CD, cluster of differentiation; COX-2, cyclooxygenase-2; Fizz1, found in inflammatory zone; IFN-γ, interferon-γ; IL, interleukin; iNOS, inducible nitric oxide synthase; MHC-II, major histocompatibility complex II; SOCS3, suppressor of cytokine signaling-3; Sphk, sphingosine kinase; SR-A1, scavenger receptor class A1; SR-B1, scavenger receptor class B1; TGF, transforming growth factor; TNF-α, tumor necrosis factor-α; Ym1, chitinase-like protein.*

**FIGURE 3:**
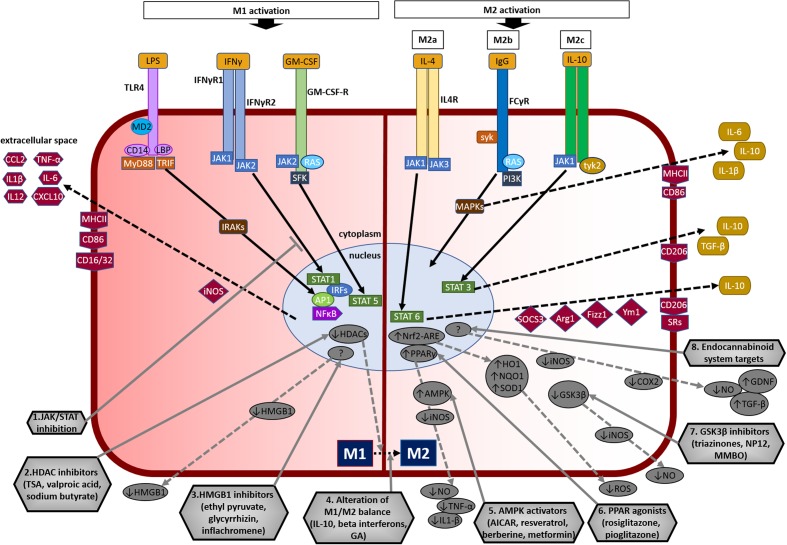
Overview of microglial M1 and M2 signaling in neurodegeneration, and potential targets for neuroprotection. The left side of the figure compartment shows M1 microglial phenotype and its major signaling pathways. LPS binds to TLR4 on the cell surface which is coupled to MD2 (TLR/MD2) with participation of co-receptors CD14 and LBP (LPS-binding protein), activates interleukin-1 receptor-associated kinases (IRAKs) through MyD88 and TRIF that causes translocation of transcription factors such as NF-κB, STAT5, activator protein-1 (AP1), and interferon regulatory factors (IRFs) to the nucleus. M1 activation by IFN-γ occurs through IFN-γ receptors 1 and 2 (IFN-γR1/2) leading to the recruitment of Janus kinase 1 and 2 (JAK1/2) that phosphorylate and translocate STAT1 and IRFs to the nucleus. M1 activation stimulation through granulocyte-macrophage colony-stimulating factor (GM-CSF) occurs when GM-CSF binds to its receptor GM-CSF-R, which activates rat sarcoma oncoproteins (RAS), JAK2, and SFK, and causes translocation of STAT5 to the nucleus. The translocation of NF-κB, STAT1, STAT5, AP1, and IRFs to the nucleus causes upregulation of intracellular iNOS and cell surface markers (CD86, CD16/32, MHC-II). M1 stimulation also causes transcriptional upregulation of M1-associated pro-inflammatory cytokines (IL-1β, IL-6, IL-12, TNF-α) and chemokines (CCL2, CXCL10). The right side of the figure compartment shows various M2 microglial phenotypes and major signaling pathways involved. M2 activation can be classified into M2a, M2b, and M2c. M2a state is induced mainly by IL-4. IL-4 binds to IL-4R, which stimulates JAK1 or JAK3 that causes translocation of STAT6 to the nucleus leading to transcription of M2a-associated genes including IL-10, cell surface markers (CD206, scavenger receptors, SRs), and intracellular components such as suppressor of cytokine signaling 3 (SOCS3), Ym1 (chitinase-like protein) and Fizz1 (found in inflammatory zone). The M2b activation state, which has some M1 response characteristics, is stimulated when TLRs fuse Fcγ receptors, which then bind to IgG (B cells) to derive the M2b phenotype. M2b activation results in secretion of IL-10 and, cell surface markers (CD86, MHC-II). M2c activation is induced by IL-10 which stimulates the IL-10 Receptor 1 and 2 subunits that activates JAK1 leading to the translocation of STAT3 to the nucleus. STAT3 translocation inhibits M1-associated pro-inflammatory cytokines and upregulation of IL-10, TGF-β and the cell surface marker CD206. M2c state plays an important role in immunoregulation, matrix deposition and tissue remodeling. The bottom half of the figure shows potential therapeutic microglial targets for neuroprotection. (1) JAK/STAT inhibition: M1 phenotype is induced via the JAK/STAT signaling pathway and inhibition of this pathway may suppress the downstream M1-associated pro-inflammatory genes. (2) Histone deacetylase (HDAC) inhibitors: Histone acetylation is increased in M1 state that may lead to the expression and release of pro-inflammatory cytokines. HDAC inhibitors prevent neurodegeneration by shifting microglia toward protective M2 phenotype and anti-inflammatory mechanisms. (3) Microglia-produced high-mobility group box-1 (HMGB1) inhibitors: HMGB1 is a pro-inflammatory cytokine released by microglia which is toxic to neurons. HMGB1 inhibitors show neuroprotection by binding to HMGB1 and inhibiting its cytokine-like activity. (4) Alteration of M1/M2 balance: These agents promote the shift M1 pro-inflammatory phenotype toward protective M2 phenotype, and also exhibit neuroprotection by counteracting excessive pro-inflammatory M1 cytokines. (5) Adenosine monophosphate-activated protein kinase (AMPK) activators: AMPK activators act by inhibiting the expression of pro-inflammatory cytokines and iNOS by reducing NF-κB activation. (6) Peroxisome proliferator-activated receptor (PPAR) agonists: PPAR agonists exhibit neuroprotection by upregulating AMPK and protective genes, reducing oxidative damage, maintaining mitochondrial function and anti-inflammatory mechanisms. (7) Glycogen synthase kinase-3 β (GSK3β) inhibitors: GSK3β regulate microglial migration, inflammation, and neurotoxicity through astrocytes. GSK3β inhibitors decrease inflammation by reducing iNOS expression and release of nitric oxide (NO). (8) Endocannabinoid system targets: Agents that target the endogenous cannabinoid ligands anandamide and 2-arachidonoylglycerol (2-AG) increase TGF-β, arginase 1 and glial cell line-derived neurotrophic factor (GDNF), and reduce iNOS and COX-2, expression. AICAR, 5-amino-4-imidazole carboxamide riboside; GA, glatiramer acetate; HO-1, heme oxygenase 1; MMBO, 2-methyl-5-methylsulfinylphenyl-1-benzofuranyl-1,3,4-oxadiazole; NP-12, tideglusib; NQO1, NAD(P)H quinone dehydrogenase 1; Nrf2, nuclear erythroid 2-related factor 2; ARE, antioxidant response element; SOD1, superoxide dismutase 1; TSA, trichostatin A.

The M1 phenotype of microglia can be induced in experimental models using microbe-associated molecular pattern molecules such as lipopolysaccharide (LPS), an endotoxin present in the cell membranes of Gram-negative bacteria, or others related to infections such as IFN-γ (**Figure 3**; Loane and Byrnes, 2010; Boche et al., 2013). Similarly, an aseptic inflammatory response also occurs after exposure to trauma or ischemia-reperfusion injury or chemical injury.

IFN-γ utilizes the JAK/STAT (Janus kinase/signal transducer and activator of transcription) signaling pathway to activate the M1 phenotype. M1 activation by IFN-γ occurs through activation of IFN-γ receptors 1 and 2 (IFN-γR1/2) leading to JAK1/2 activation, phosphorylation and the nuclear translocation of STAT1 along with interferon regulatory factors (IRFs). This signaling cascade promotes expression of M1-associated cytokines, chemokines, and other genes (Hu and Ivashkiv, 2009; Boche et al., 2013).

LPS induces M1 activation via TLRs, which recognize specific patterns of microbial macromolecules. LPS binds to TLR4 on the cell surface that is coupled to MD2 (myeloid differentiation protein 2) (TLR/MD2) with participation of co-receptors CD14 and LBP (LPS-binding protein). LPS binding to TLR4 results in activation through MyD88 (myeloid differentiation primary response protein 88) and TRIF (TIR domain-containing adaptor inducing IFN-β), and transcription factors such as NF-κB (nuclear factor kappa B), STAT5, and IRFs (Takeda and Akira, 2004). This causes transcriptional upregulation of M1-associated cytokines, chemokines and other genes. Alternative M1 activation stimulation through granulocyte-macrophage colony-stimulating factor (GM-CSF) has been demonstrated recently (Lacey et al., 2012). However, unlike LPS and IFN-γ, GM-CSF is reported to instigate pleomorphic activation states that shows characteristics of both M1 and M2 phenotypes (Weisser et al., 2013). **Figure 3** (left side) provides further details on M1 activation.

### M2 Polarization State

The alternative M2 microglial activation state is involved in various events including immunoregulation, inflammation dampening, and repair and injury resolution. M2 microglia is morphologically characterized by enlarged cell bodies (**Figure 2**). M2 microglial activation produces an array of mediators such as anti-inflammatory cytokines, extracellular matrix proteins, glucocorticoids, and other substances.

Presently, the mechanism of M2 activation in microglia is poorly understood compared to macrophages. It is believed that microglia can develop diverse M2 phenotypes similar to macrophages (Morgan et al., 2005; Herber et al., 2006; Schwartz et al., 2006). The characteristics of M2 polarization of microglia parallel that of the macrophages (Chhor et al., 2013; Freilich et al., 2013) producing IL-4 and IL-10 stimulation through Arg1 (arginase 1), Ym1 (chitinase-like protein), Fizz1 (found in inflammatory zone), and PPAR (peroxisome proliferator-activated receptor) (Michelucci et al., 2009). M2 macrophage activation is sub-classified into M2a, M2b, and M2c, and these activation states may be envisaged to microglia (Boche et al., 2013; **Figure 3** and **Table 1**). The M2a state is induced by IL-4 or IL-13 and, is associated with tissue repair and phagocytosis. IL-4 binds to different receptor pairs, which stimulate JAK1 or JAK3 and activates STAT6 leading to transcription of M2a-associated genes including CD206, suppressor of cytokine signaling 3 (SOCS3) and SRs. The M2b activation state is stimulated by engagement of TLRs and the IL-1 receptor, and is involved in the recruitment of regulatory T cells. Activated TLRs fuse Fcγ receptors, which then bind to IgG (B cells) to derive the M2b phenotype. M2b activation results in secretion of IL-10, CD86 (on cell surface) and MHC-II. M2c activation state is induced by IL-10 and glucocorticoid hormones, and is involved in anti-inflammatory and healing functions. IL-10 stimulates the IL-10R1 and IL-10R2 which activate JAK1 leading to the translocation of STAT3 into the nucleus. STAT3 translocation suppresses most of the M1-associated pro-inflammatory cytokines (Franco and Fernandez-Suarez, 2015; Michell-Robinson et al., 2015). Overall, it is considered that the M2 activation promotes healing and tissue repair whereas the M1 activation state is the first line of defense with pro-killing functions. Please see **Figure 3** (right side) for details on M2 activation.

### Polarization Transitions

The transition from the M1 pro-inflammatory state to the regulatory or anti-inflammatory M2 phenotype is thought to assist improved functional outcomes and restore homeostasis (Orihuela et al., 2016). Recently, histone H3K27me3 demethylase Jumonji was shown to be essential for M2 polarization and down-regulation of the M1 phenotype (Tang et al., 2014). The induction of M1 phenotype is a relatively standard response during injury. For peripheral immune cells it is thought that M1 polarization is terminal and the cells die during the inflammatory response (Orihuela et al., 2016). Although a shift from M1 to the M2 phenotype is considered rare for peripheral immune cells, the microglia *can shift* from M1 to M2 phenotype when exposed to IL-10, glatiramer acetate, beta interferons, PPARγ agonists and other molecules discussed in the later section. Although, the M1 and M2 microglial phenotypes vastly differ in their function, different subpopulations in an injury environment may express specific phenotypes resulting in concurrent expression of M1- and M2-related factors or mixed M1/M2 phenotypes (Ziegler-Heitbrock et al., 2010; Pettersen et al., 2011; Vogel et al., 2013). The potential to pharmacologically promote a microglial M1 to M2 shift may have therapeutic implications in the setting of neurodegenerative diseases associated with neuroinflammation.

## Microglia-Mediated Inflammation in PD

The involvement of innate immunity in PD was first proposed by McGeer et al. (1988) when brain from PD patients showed high levels of reactive microglia that were positive for human leukocyte antigen-D related (HLA-DR) in the substantia nigra and putamen. GWAS indicate that variants in the HLA region are linked to sporadic PD (Hamza et al., 2010; Hill-Burns et al., 2011). Activated microglia in PD brain appear responsible for exacerbating neurodegeneration (McGeer and McGeer, 2004), and the exposure of human neuromelanin discharged from dead DA neurons cause chemotaxis and increases the pro-inflammatory substances in microglial cultures (Wilms et al., 2007). M1 activation-associated inflammatory markers such as MHC-II (Imamura et al., 2003), TNF-α and IL-6 (Boka et al., 1994; Imamura et al., 2003) have been reported in patients with PD. Recent positron emission tomography (PET) studies show that PD patients have cortical microglial activation and lower brain glucose metabolism early in the disease, and imply that microglial activation may be a contributing factor in the disease progression (Edison et al., 2013). PET with inflammatory ligands show elevation in several areas of the basal ganglia involved in PD pathology (Gerhard et al., 2006; Edison et al., 2013; Iannaccone et al., 2013). TLR2 is increased in postmortem PD brain tissue, which correlates with pathological α-synuclein deposition. The neuronal TLR2 rather than glial expression of TLR2 is significantly elevated in PD brain and correlates with disease progression. In addition, TLR2 is strongly localized in α-synuclein positive Lewy bodies (Dzamko et al., 2017). These observations highlight the crucial role of neuroinflammation in PD pathogenesis.

## Peripheral Inflammation in PD

“The dual hit theory” of PD development states that a neurotropic pathogen enters the brain by nasal and/or gastric route by axonal transport, the latter via the vagus nerve (Braak et al., 2003; Hawkes et al., 2009). There is evidence that some forms of α-synuclein can be transmitted from the gut to the brain (Pan-Montojo et al., 2010; Ulusoy et al., 2013; Holmqvist et al., 2014). Instillation of rotenone into the rodent stomach exhibits progressive pathological α-synuclein inclusions in the enteric nervous system, the vagus nerve and subsequently in the brain stem (Pan-Montojo et al., 2010). Vagotomy prevents transport of pathological proteins from the gut to CNS (Phillips et al., 2008; Pan-Montojo et al., 2012). A recent study in Danish patients reveals that those who underwent full truncal vagotomy had lower risk for PD, suggesting that the vagus nerve might be critically involved in PD pathogenesis (Svensson et al., 2015). Another clinical study reports that serum levels of the pro-inflammatory cytokine IL-1β discriminated asymptomatic LRRK2-G2019S carriers from controls and suggests that peripheral inflammation is greater in a percentage of subjects carrying LRRK2-G2019S mutation (Dzamko et al., 2016).

The major peripheral immune cells, T-lymphocytes and B-lymphocytes, are not found in the CNS in normal biological conditions. However, with peripheral inflammation such as infection or injury, blood monocytes, and tissue-resident immune cells are activated and secrete variety of pro-inflammatory mediators including TNF-α, IL-6, and IL-1β. These pro-inflammatory mediators cross the blood–brain barrier leading to the activation of brain resident microglia, which then triggers a neuroinflammatory cascade. The blood–brain barrier is considered to be impermeable to external pathogens and circulating macrophages, hence serving as an additional line of defense to the CNS. Nevertheless, damage to the integrity of the blood–brain barrier renders the brain vulnerable. PET studies on patients with PD reveal dysfunctional blood–brain barrier (Kortekaas et al., 2005). Damage to the blood–brain barrier in rats induce degeneration of dopaminergic neurons in the substantia nigra and activate glial cells (Rite et al., 2007). CD8^+^ and CD4^+^ T cells are observed in the postmortem human PD brain and MPTP (1-methyl-4-phenyl-1,2,3,6-tetrahydropyridine) mouse model of PD during its neurodegenerative phase suggesting T cell-mediated dopaminergic toxicity as a putative mechanism (Brochard et al., 2009). Moreover, rats with ulcerative colitis are more susceptible to LPS-induced dopaminergic neuron loss, blood–brain barrier permeability, microglial activation, and generation of pro-inflammatory mediators suggesting that peripheral inflammation may increase the risk of PD (Villaran et al., 2010). A recent study in the acute MPTP mouse model where nigrostriatal pathologies are not robust show that administration of chemokines [regulated on activation, normal T cell expressed and secreted (RANTES) and eotaxin] that facilitate T cell trafficking can lead to marked nigral α-synuclein pathology, loss of dopaminergic neurons and striatal neurotransmitter depletion, glial-associated inflammation, and motor impairments. However, systemic administration of pro-inflammatory cytokines, TNF-α and IL-1β, did not induce such disease pathologies in this acute MPTP model (Chandra et al., 2017). Taken together these studies suggest a more direct link between peripheral inflammation and, potential to elicit and affect the timing of PD onset.

## Genetic Factors Linked to Inflammation in PD

Mutations in leucine-rich repeat kinase (LRRK2, PARK8) are linked with autosomal dominantly inherited PD and is the greatest known genetic contributor to PD. LRRK2-G2019S mutation in the kinase domain is the most common mutation in both familial and sporadic form of the disease (Goldwurm et al., 2005). GWAS show that the genetic locus containing the LRRK2 gene presents a risk factor for sporadic PD (Singleton and Hardy, 2011). Interestingly, GWAS implicate LRRK2 as a major susceptibility gene in inflammatory bowel diseases that involve chronic inflammation (Barrett et al., 2008; Liu and Lenardo, 2012). LRRK2 is reported to be a target gene for IFN-γ, a M1-activation-associated pro-inflammatory cytokine. LRRK2 expression is elevated in human intestinal tissue of patients with Crohn’s disease inflammation (Gardet et al., 2010). LRRK2 shows high expression in immune cells including microglia and inhibition of LRRK2 function reduces M1-associated inflammation and PD neurodegeneration (Moehle et al., 2012; Daher et al., 2014, 2015; Russo et al., 2015). Collectively, these studies point toward the importance of LRRK2 function in M1-activation responses in PD animal models (Moehle and West, 2015).

Three different missense mutations (A530T, A30P, and E46K) within the open reading frame or duplication or triplication of the wild type α-synuclein gene (SNCA, PARK1) are associated with autosomal dominant PD (Polymeropoulos et al., 1997; Kruger et al., 1998; Singleton et al., 2003; Zarranz et al., 2004). Fibrillar forms of α-synuclein are a major component of the Lewy bodies in both sporadic and familial PD. α-Synuclein, a cytoplasmic protein, can be expressed in microglia and may be involve modulation and pre-sensitization of microglial activation (Barkholt et al., 2012; Roodveldt et al., 2013; Zhang et al., 2017). Most activated microglia in PD patient brains are associated with α-synuclein-positive Lewy neurites (Imamura et al., 2003), and there is significant correlation between the expression of M1 activation-associated marker MHC-II and α-synuclein deposition in the substantia nigra of PD patients (Croisier et al., 2005). Previous studies by our group and others using *in vitro* and animal models show that both wild type and mutant α-synuclein can modulate microglial activation leading to neuroinflammation. Our previous work shows that α-synuclein activates microglia in a dose-dependent manner in cultured cells and an early microglial activation event occurs in mice overexpressing wild type α-synuclein (Su et al., 2008). Another study reveals that mutant α-synuclein can directly interact with cultured microglia releasing pro-inflammatory substances and mice overexpressing mutant α-synuclein exhibit microglial activation at a very early age (Su et al., 2009). In addition, we show that misfolded α-synuclein induces microglial activation and the release of pro-inflammatory cytokines in BV2 microglial cells (Beraud et al., 2013). Overexpression of α-synuclein in BV2 microglial cells increase pro-inflammatory mediators (TNF-α, IL-6, nitric oxide, COX-2) and induce a reactive microglial phenotype (Rojanathammanee et al., 2011). Interestingly, TLR4, which is activated by LPS to induce microglial M1-phenotype, is reported to mediate α-synuclein-induced microglial phagocytosis, upregulation of pro-inflammatory cytokine expression and ROS generation in primary microglial cultures (Fellner et al., 2013). In addition, α-synuclein is reported to play a crucial role in modulating microglial activation states in postnatal brain derived microglial cultures (Austin et al., 2006). Moreover, neuroinflammation with activated microglia and increased pro-inflammatory cytokines (TNF-α, IL-1β, IFN-γ) precedes α-synuclein-mediated neuronal cell death in rats delivered with mutant A53T human α-synuclein (Chung et al., 2009). There is a rich literature on the role of α-synuclein in the progression of PD by inducing microglia activation and neuroinflammation which is reviewed elsewhere (Zhang et al., 2017).

In addition, genes linked to familial recessive PD including phosphatase and tensin homolog (PTEN)-induced kinase 1 (PINK1, PARK6) and DJ-1 (PARK7) are strongly associated with neuroinflammation. Deletion of PINK1 or DJ-1 causes aberrant expression of genes involved in p38 MAP kinase/NF-κB signaling pathways that regulate innate immune responses in disease models (Cornejo Castro et al., 2010; Akundi et al., 2011).

## Inflammation in PD Animal Models

LPS, a bacterial endotoxin from cell wall of Gram-negative bacteria, induces M1-polarization of microglia through activation the pattern recognition TLR4. LPS administration into rodent brains recapitulate certain characteristics of sporadic PD including progressive degeneration of nigrostriatal dopaminergic pathway and motor anomalies. A single injection or 2-week infusion of LPS in the supranigral region in rat brain causes rapid microglia activation followed by dose and time-dependent degeneration of nigrostriatal dopaminergic circuitry (Castano et al., 1998; Gao et al., 2002; Liu, 2006; Dutta et al., 2008). Direct injection of LPS shows dopaminergic neuron loss specifically in SNpc but not in ventral tegmental area which also houses dopaminergic neurons (Kim et al., 2000). This specific neurotoxicity in SNpc may be attributed to the high proportion of microglia in SNpc compared with other brain regions (Lawson et al., 1990), that may trigger inflammatory events leading to the degeneration of nigrostriatal pathway. Moreover, injection of a TLR3 agonist in the substantia nigra of adult rats induces a sustained inflammatory reaction in the substantia nigra (SN) and dorsolateral striatum, and also increases the vulnerability of midbrain dopaminergic neurons to a subsequent neurotoxic trigger (Deleidi et al., 2010).

A transgenic mouse PD model that overexpresses human wild type α-synuclein, Thy1-aSyn (line 61) (Chesselet et al., 2012), shows microglial activation as early as 1 month and persists until 14 months of age (Watson et al., 2012). Increased levels of TNF-α, TLRs (TLR1, TLR2, TLR4, and TLR8), MHC-II, CD4, and CD8 are observed in Thy1-aSyn mice at different ages. This study also reveals that despite expression of α-synuclein globally in the brain only the regions containing cell bodies and axon terminals of nigrostriatal pathway show early inflammatory response. Another transgenic rodent model overexpressing doubly mutated (A53T and A30P) human α-synuclein show glial mitochondria alterations (Schmidt et al., 2011). In a PD mouse model that overexpresses human α-synuclein by recombinant adeno-associated virus vector, serotype 2 (rAAV2)-mediated transduction in the SNpc, inflammatory responses such as microglial activation and greater infiltration of B and T lymphocytes are observed in addition to dopaminergic neurodegeneration (Theodore et al., 2008). These studies show that animal models overexpressing human or mutant α-synuclein exhibit microglial activation and neuroinflammation.

MPTP, a meperidine analog byproduct, is a neurotoxin that causes acute and irreversible human parkinsonism (Davis et al., 1979; Langston et al., 1983). MPTP is a lipophilic compound that can actively cross the blood–brain barrier and gets oxidized by monoamine oxidase to the toxic cation, MPP^+^ (1-methyl-4-phenylpyridinium) in the glial cells (Langston et al., 1984; Markey et al., 1984). MPP^+^ utilizes the DA transporter (DAT) to get into the dopaminergic neurons. MPP^+^ accumulates in the mitochondria and inhibits the mitochondrial complex I in the electron transport chain (ETC) (Nicklas et al., 1985; Ramsay et al., 1986) resulting in reduced ATP levels and production of ROS (Hasegawa et al., 1990; Chan et al., 1991; Hantraye et al., 1996; Przedborski et al., 1996; Fabre et al., 1999; Pennathur et al., 1999). In animal models, MPTP induces inflammatory responses that lead to neurodegeneration. MPTP causes microglial activation and an increase in M1-associated pro-inflammatory cytokines such as IL-6, IFN-γ and TNF-α. The glial response to MPTP is reported to peak before dopaminergic neuron loss (Czlonkowska et al., 1996; Smeyne and Jackson-Lewis, 2005). In support of these findings, it is reported that mice lacking IFN-γ or TNF-α signaling show resistance to MPTP-induced neurodegeneration (Sriram et al., 2002; Mount et al., 2007). Mice treated with MPTP show T-cell (CD4^+^) infiltration into the substantia nigra and the MPTP-induced dopaminergic neuron loss is attenuated in T-cell deficient mice suggesting a pro-inflammatory role for T-cells (CD4^+^) in MPTP neurotoxicity (Brochard et al., 2009). In addition, treatment with anti-inflammatory agents such as minocycline (Du et al., 2001), ibuprofen (Swiatkiewicz et al., 2013), flavonoid pycnogenol (Khan et al., 2013) and peptide carnosine (Tsai et al., 2010) and inhibition of pro-inflammatory mediators (Watanabe et al., 2004; Zhao et al., 2007), reduce inflammation and prevent neurodegeneration in MPTP animal models.

## Regulators of Microglial Activation States

Microglial activation, astrogliosis and invasion of activated peripheral immune cells trigger deleterious events in the brain that lead to neuronal loss and progression of PD (Hirsch and Hunot, 2009). These observations led to several animal studies and clinical trials to test a variety of established anti-inflammatory molecules (see **Table 2**). Acetylsalicylic acid, a COX-1/COX-2 inhibitor, exhibits neuroprotective effects in *in vitro* and in MPTP animal models of PD (Teismann and Ferger, 2001). A prospective clinical study shows that consumption of non-aspirin NSAIDs may delay or prevent the onset of PD (Chen et al., 2003). A Cochrane collaboration study which analyzed several prevention trials and observational anti-inflammatory studies reveals that while ibuprofen use might reduce the risk of developing PD, there is no existing evidence that supports NSAID use in PD prevention (Rees et al., 2011). A clinical study in PD cases shows an association between use of ibuprofen and lesser PD risk. However, this association is not shared by other NSAIDs studied (Gao et al., 2011). Similarly, minocycline, a tetracycline antibiotic that showed promising anti-inflammatory properties in PD animal models (Du et al., 2001; He et al., 2001; Wu et al., 2002), did not provide any symptomatic improvement in PD patients (Ninds Net-Pd Investigators, 2008). See **Table 2** for the list of anti-inflammatory agents studied in PD. Hence, NSAID use appears to provide benefits in PD susceptibility in some cases (Wahner et al., 2007; Samii et al., 2009; Gagne and Power, 2010; Steurer, 2011), however, this beneficial effect of NSAIDs were not replicated in several other studies (Shaunak et al., 1995; Bornebroek et al., 2007; Samii et al., 2009; Becker et al., 2011). One study even suggests that anti-inflammatory drug treatment may be detrimental if given at the later stage of the disease (Keller et al., 2011). This therapeutic approach aiming to counteract general neuroinflammation has failed in several disease therapies as reviewed elsewhere (Pena-Altamira et al., 2016). Collectively, these studies indicate that the non-specific inflammatory blockade is unlikely to be beneficial for the disease treatment. Concurrently, the data on the dual role of microglial activation has led to the emergence of the novel therapeutic strategy in other inflammatory diseases such as rheumatoid arthritis, ankylosing spondylitis, and multiple sclerosis (MS) (Rau, 2002; Braun et al., 2007; Wilms et al., 2010; Noda et al., 2013). This approach of the M1 to the M2 phenotype shift might be beneficial in neuroprotection compared to completely blocking microglial activation through anti-inflammatory drugs. Hence, a more reasonable approach of more specific treatment related to the M1/M2 activation stage by inhibiting the M1 responses and/or promoting the shift of M1 to M2 phenotypic responses is necessary in PD.

**Table 2:** NSAIDs and other anti-inflammatory agents in PD models and clinical trials.

Molecule	PD model	Mechanism	Response	Reference
Ibuprofen	Mouse MPTP	Anti-inflammatory	Prevent striatal-TH loss	Swiatkiewicz et al., 2013
	Clinical trial	Anti-inflammatory	Reduce PD risk	Gao et al., 2011

Acetylsalicylic acid	Mouse MPTP	COX-1/COX-2 inhibitor	Attenuate loss of nigral DA-neurons, striatal dopamine and locomotor activity	Teismann and Ferger, 2001

NSAIDs	Clinical trials	Anti-inflammatory	Delay or prevent onset of PD	Chen et al., 2003; Wahner et al., 2007; Samii et al., 2009; Gagne and Power, 2010; Steurer, 2011
	Clinical trials	Anti-inflammatory	Exacerbate PD symptoms/Does NOT improve PD risk	Shaunak et al., 1995; Bornebroek et al., 2007; Samii et al., 2009; Becker et al., 2011

Minocycline	Mouse MPTP Mouse 6-OHDA	Anti-inflammatory Anti-inflammatory	Attenuate loss of nigral DA-neurons and striatal dopamine Protect TH-positive cells	Du et al., 2001; Wu et al., 2002; He et al., 2001
	Clinical trial	Anti-inflammatory	Does NOT improve PD symptoms	Ninds Net-Pd Investigators, 2008


*COX, cyclooxygenase; DA, dopamine; MPTP, 1-methyl-4-phenyl-1,2,3,6-tetrahydropyridine; NSAIDs, non-steroidal anti-inflammatory drugs; TH, tyrosine hydroxylase.*

### Targeting M1 Polarization State: Inhibition of Pro-inflammatory M1 Phenotype

M1 activation of microglia results in a pro-inflammatory and pro-killing output. To inhibit the pro-inflammatory damage through M1 activation of microglia, its downstream signaling pathways could be targeted. M1 phenotype is induced by IFN-γ via the JAK/STAT signaling pathway and targeting this pathway may arrest M1 activation. In fact, studies show that inhibition of the JAK/STAT pathway leads to suppression of the downstream M1-associated genes in several disease models including experimental allergic encephalomyelitis models and myeloproliferative neoplasms (Liu et al., 2014; Mascarenhas et al., 2014). Another approach to suppress M1 activation would be to target the pro-inflammatory cytokines such as TNF-α, IL-1β and IFN-γ, and decrease its ability to interact with its receptors on other cell types. TNF has been targeted through different approaches in PD animal models to suppress M1-associated toxicity. A single injection of lentivirus-expressing dominant negative TNF (DN-TNF) into the rat substantia nigra concomitantly with 6-hydroxydopamine (6-OHDA) lesion in the striatum attenuates dopaminergic neuron loss and behavioral anomalies in rats (McCoy et al., 2008). In another study intended to examine the role of TNF in delayed and progressive neurodegeneration model, rats administered with DN-TNF in the substantia nigra 2 weeks after the 6-OHDA lesion show no further dopaminergic neuron loss even after 5 weeks of 6-OHDA suggesting that TNF is an essential mediator of inflammation and hence a promising therapeutic target in PD (Harms et al., 2011). In addition, adeno-associated virus (AAV)-mediated transduction of dopaminergic neurons with human ras homolog enriched in brain with S16H mutation, [hRheb(S16H)], attenuates nigrostriatal toxicity in 6-OHDA rat model of PD (Kim et al., 2011, 2012). This protective effect is mediated by the production of cAMP response element-binding protein (p-CREB), glial cell line-derived neurotrophic factor (GDNF), and brain-derived neurotrophic factor (BDNF) in unilateral MPP^+^ neurotoxin PD model (Nam et al., 2015).

PPARs are actively involved in microglial activation and inflammatory pathways. PPAR agonists are postulated to be beneficial for PD and other neurodegenerative diseases (Agarwal et al., 2017). The administration of a PPARγ agonist, rosiglitazone, arrests degeneration in both striatum and SNpc by decreasing TNF-α production and modulating microglial polarization in MPTPp (MPTP + probenecid) progressive mouse model of PD (Carta et al., 2011; Pisanu et al., 2014). Pioglitazone, a PPARγ agonist, prevents tyrosine hydroxylase (TH)-positive neuron loss in substantia nigra and partially averts striatal DA loss in MPTP mice model of PD. Pioglitazone treatment decreases microglial activation, iNOS production and nitric oxide-mediated toxicity in both striatum and substantia nigra (Dehmer et al., 2004). However, a recent clinical trial concluded that pioglitazone did not modify progression in early PD (Ninds Exploratory Trials in Parkinson Disease (Net-Pd) Fs-Zone Investigators, 2015). On the other hand, pioglitazone and rosiglitazone are currently being evaluated in clinical trials for its potential to reduce progression of AD. In addition, treatment of LPS/IFN-γ-activated neuronal and glial cultures with a PPARγ endogenous ligand, 15-deoxy-Δ^12,14^-prostaglandin J_2_, inhibits pro-inflammatory response through the CD200-CD200R1 dependent mechanism (Dentesano et al., 2014). Unpublished data from our group show that administration of a PPAR agonist protects dopaminergic neurons in SNpc and neurites in striatum in MPTP mouse model of PD. This PPAR agonist reduces LPS-induced M1-associated pro-inflammatory cytokine IL-1β in BV2 cells and primary astrocytes in a PPARα-independent manner. Thus, PPAR agonists are potential molecules for curing PD through their property to inhibit M1 microglia-induced neuroinflammation.

Alterations in expression of cannabinoid receptors and endocannabinoid concentrations are illustrated in PD pathogenesis (Garcia et al., 2015). The endocannabinoid system includes the cannabinoid receptors CB1 and CB2, the endogenous ligands (anandamide and 2-arachidonoylglycerol, 2-AG), and their regulatory enzymes. CB1 receptors are abundant in neurons whereas CB2 receptors are most specifically expressed in glia (Onaivi, 2006; Lanciego et al., 2011; Fernandez-Suarez et al., 2014; Sierra et al., 2015). The expression of CB2 receptors significantly increases during microglial activation (Maresz et al., 2005; Yiangou et al., 2006) and CB2 receptors are reported to be localized in substantia nigra, and significantly downregulated in PD patients (Garcia et al., 2015). A naturally occurring CB2 receptor agonist, β-caryophyllene (BCP), prevented nigral DA-neuron and striatal-TH loss, reduced glial activation and inhibited pro-inflammatory cytokines in rat rotenone model of PD (Javed et al., 2016). Another study shows that a non-selective cannabinoid agonist protects loss of DA-neurons in the substantia nigra and DA in the striatum of MPTP intoxicated mice. In addition, this cannabinoid agonist also reduces MPTP-induced motor deficits and microglial activation (Price et al., 2009). Modification of the endocannabinoid system, to reduce pro-inflammatory toxicity, may provide a novel therapeutic avenue for PD treatment.

Tanshinone-I, a bioactive flavonoid, reduces the production of M1-pro-inflammatory mediators (nitric oxide, TNF-α, IL-6 and IL-1β) and, inhibits G-CSF and NF-κB expression after LPS-induced inflammation in BV2 microglial cell lines. In the MPTP mouse model of PD, Tanshinone-I prevents dopaminergic neurotoxicity, improves motor deficits and striatal neurotransmitters (Wang et al., 2015). Ghrelin, a stomach-derived endogenous ligand for growth hormone secretagogue receptor 1a (GHS-R1a), prevents loss of nigral dopaminergic neurons and striatal neurites, and improves motor performance in MPTP mouse model of PD. Ghrelin reduces toxicant-induced microglial activation, production of pro-inflammatory cytokines (IL-1β, TNF-α) and iNOS levels in MPTP mice (Jiang et al., 2008; Moon et al., 2009). Piperine, a naturally occurring bioactive molecule, attenuates the loss of TH-positive neurons in the substantia nigra and MPTP-induced motor anomalies. In addition, piperine decreases MPTP-induced microglial activation, pro-inflammatory IL-1β expression and apoptosis in these mice (Yang et al., 2015). See **Table 3** for the summary of other potential molecules that act by inhibiting M1 activation in PD models.

**Table 3:** Molecules targeting microglia activation in PD animal models and clinical trials.

Molecule	PD model	Mechanism	Response	Reference
Pioglitazone	Mouse MPTP	Inhibit M1 phenotype	Prevent loss of nigral DA-neurons and partial striatal neurites	Dehmer et al., 2004
	Clinical trial	Inhibit M1 phenotype	Does NOT modify progression in early PD	Investigators, 2015

Rosiglitazone	Mouse MPTPp (MPTP + probenecid)	Inhibit M1 phenotype and induce M2 phenotype	Prevent loss of nigral DA-neurons and striatal neurites	Carta et al., 2011; Pisanu et al., 2014

Lentivirus-expressing dominant negative TNF (DN-TNF)	Rat 6-OHDA (concomitant model)	Inhibit M1-TNF	Prevent nigral DA-neuron loss and behavior deficits	McCoy et al., 2008

Lentivirus-expressing DN-TNF	Rat 6-OHDA (delayed model)	Inhibit M1-TNF	Prevent progressive nigral DA-neuron loss	Harms et al., 2011

AAV-expressing human IL-10	Mouse MPTP	Induce M2 phenotype	Prevent loss of striatal dopamine and TH	Schwenkgrub et al., 2013; Joniec-Maciejak et al., 2014

Glatiramer acetate	Mouse MPTP	Induce M2 phenotype	Prevent loss of nigral DA-neurons	Benner et al., 2004; Burger et al., 2009

Endocannabinoid ligand 2-AG enhancer	Mouse MPTPp	Induce M2 phenotype	Prevent loss of striatal dopamine and TH	Fernandez-Suarez et al., 2014

Tanshinone-I	Mouse MPTP	Inhibit M1 phenotype	Prevent nigral DA-neuron loss and motor deficits	Wang et al., 2015

β-Caryophyllene	Rat-rotenone	Inhibit M1 phenotype	Prevent loss of nigral DA-neurons and striatal neurites	Javed et al., 2016

Atractylenolide-I	Mouse MPTP	Inhibit M1 phenotype	Attenuate loss of nigral DA-neurons and behavior deficits	More and Choi, 2017

Loganin	Mouse MPTP	Inhibit M1 phenotype	Prevent loss of striatal dopamine and TH	Xu et al., 2017

α-Asarone	Mouse MPTP	Inhibit M1 phenotype	Attenuate behavior deficits	Kim et al., 2015

Capsaicin	Mouse MPTP	Inhibit M1 phenotype	Attenuate loss of nigral DA-neurons, striatal dopamine, and behavioral deficits	Chung et al., 2017

Isobavachalcone	Mouse MPTP	Inhibit M1 phenotype	Attenuate behavior deficits and neuronal necrosis	Jing et al., 2017

Vitamin-D	Mouse MPTP	Inhibit M1 phenotype and induce M2 phenotype	Prevent loss of TH-positive neurons	Calvello et al., 2017

Mitoapocynin	Mouse MPTP	Inhibit M1 phenotype	Attenuate loss of nigral DA-neurons, striatal dopamine and behavioral deficits	Ghosh et al., 2016

Ginsenoside Rg1	Mouse MPTPp	Inhibit M1 phenotype	Attenuate loss of nigral DA-neurons and behavior deficits	Heng et al., 2016


*AAV, adeno-associated virus; DA, dopamine; IL, interleukin; MPTP, 1-methyl-4-phenyl-1,2,3,6-tetrahydropyridine; 6-OHDA, 6-hydroxydopamine; TH, tyrosine hydroxylase; TNF, tumor necrosis factor.*

### Targeting M2 Polarization State

The molecules with the capability to activate the anti-inflammatory M2 phenotype or promote the transition of pro-inflammatory M1 phenotype to anti-inflammatory M2 could be useful in the treatment of PD. Anti-inflammatory molecules such as IL-10 and beta interferons produce neuroprotection by altering the M1 and M2 balance. Cerebral infusion of AAV-expressing human IL-10 in a MPTP mouse model of PD decreases the expression of pro-inflammatory iNOS and importantly enhances the levels of anti-inflammatory mediators including IFN-γ and transforming growth factor-β (TGF-β). Infusion of AAV-expressing human IL-10 prevents the loss of striatal DA and reduces TH transcriptome levels suggesting neuroprotection in MPTP intoxicated mice (Schwenkgrub et al., 2013; Joniec-Maciejak et al., 2014).

Treatment with pioglitazone, a PPARγ agonist, causes a phenotypic conversion of microglia from the pro-inflammatory M1 state to the anti-inflammatory M2 state. This conversion is strongly linked to increase in phagocytosis of misfolded protein deposits resulting in the reduction of amyloid levels and an associated reversal of contextual memory deficits in AD mice (Mandrekar-Colucci et al., 2012). As mentioned before, pioglitazone treatment decreases microglial activation, iNOS production and NO-mediated nigrostriatal toxicity in MPTP mouse model (Dehmer et al., 2004).

The endocannabinoid system is implicated in PD pathogenesis (Garcia et al., 2015). In a chronic MPTPp model of PD, the administration of an inhibitor that prevents degradation of 2-AG (JZL184), an endocannabinoid ligand, prevents MPTPp-induced motor impairment and protects the nigrostriatal pathway (Fernandez-Suarez et al., 2014). MPTPp mice treated with JZL184 exhibits microglial phenotypic changes and restorative microglial activation along with increased TGF-β and GDNF levels.

Glatiramer acetate is a Food and Drug Administration (FDA) approved drug for MS treatment (English and Aloi, 2015) and its neuroprotective effect is mediated by activation of the microglial M2 phenotype (Giunti et al., 2014). Glatiramer acetate protects dopaminergic neurons in a MPTP mouse model by helping the recruitment of T lymphocytes in the SN, while inhibiting microglial activation and upregulation of GDNF expression (Benner et al., 2004; Burger et al., 2009). Glatiramer acetate also exhibits neuroprotection in Alzheimer’s disease animal models where its treatment induces microglial co-localization with amyloid fibrils and a switch in microglial phenotype that produces insulin like growth factor 1 (Butovsky et al., 2006). Dimethyl fumarate (DMF), an approved drug for MS treatment, protects against the depletion of striatal DA and its transporters and, reduces MPTP-induced increase in IL-1β and COX-2 activity in MPTP mouse model of PD. DMF also modulates microglial activation states and restores nerve growth factor levels to provide neuroprotection in MPTP-intoxicated mice (Campolo et al., 2017). Other molecules that are reported to possess neuroprotective properties by inducing M2 microglial activation are listed in **Table 3**.

## Novel Microglial Targets

Other novel potential microglial targets for immunomodulation are reviewed elsewhere (Pena-Altamira et al., 2016). These approaches include the following targets: (1) 5′ adenosine monophosphate-activated protein kinase (AMPK): a critical enzyme in cellular energy homeostasis, (2) microglia-produced high-mobility group box-1 (HMGB1): an early released pro-inflammatory cytokine, (3) glycogen synthase kinase-3β (GSK3β): an enzyme that mediates microglial migration and inflammation-induced neurotoxicity, and (4) histone deacetylases (HDACs).

## Concluding Remarks and Future Directions

The critical role of microglia in most neurodegenerative pathologies including PD is increasingly documented through many studies. Until recently, microglial activation in pathological conditions was considered to be detrimental to neuronal survival in the substantia nigra of PD brains. Recent findings highlight the crucial physiological and neuroprotective role of microglia and other glial cells in neuropathological conditions. Studies on anti-inflammatory treatments targeting neuroinflammation in PD and other diseases by delaying or blocking microglial activation failed in many trials due to the lack of a specific treatment approach, possibly the stage of disease and an incorrect understanding of mechanisms underlying microglial activation. With the updated knowledge on different microglial activation states, drugs that can shift microglia from a pro-inflammatory M1 state to anti-inflammatory M2 state could be beneficial for PD. The M1 and M2 microglial phenotypes probably need further characterization, particularly in PD pathological conditions for better therapeutic targeting. We support targeting of microglial cells by modulating their activation states as a novel therapeutic approach for PD.

## Author Contributions

HF conceived of the project, SS and HF wrote the manuscript.

## Conflict of Interest Statement

The authors declare that the research was conducted in the absence of any commercial or financial relationships that could be construed as a potential conflict of interest.
